# Spatiotemporal Double‐Edged Sword of Macrophages: Temporal Regulation of Neuroinflammation and Neurorepair in Ischemic Stroke

**DOI:** 10.1155/jimr/1172895

**Published:** 2026-05-03

**Authors:** Shuwen Zhang, Xinyu Chai, Shanzhu Guo, Yuxi Wang, Yinan Wang, Hongyan Yuan, Dongmei Yan

**Affiliations:** ^1^ Department of Immunology, Jilin University, Changchun, China, jlu.edu.cn; ^2^ Key Laboratory of Pathobiology, Ministry of Education, Jilin University, Changchun, 130021, China, jlu.edu.cn

**Keywords:** ischemic stroke, macrophages, neural repair, neuroinflammation, phenotypic polarization

## Abstract

Ischemic stroke (IS) accounts for ~85% of all stroke cases and stands as one of the leading global causes of disability and mortality. Its pathological progression is closely intertwined with intricate inflammatory responses, among which post‐stroke activated macrophages are widely recognized as the core drivers and regulators of IS pathogenesis. This review systematically elucidates the dual role of macrophages in stroke: they act as the primary drivers of the acute neuroinflammatory storm, while also serving as key regulators of neurorepair during the subacute and chronic phases. By focusing on the spatiotemporal dynamic changes, polarization regulatory mechanisms, and phenotypic/functional transition patterns of macrophages. This review provides a theoretical foundation for the development of precise therapeutic strategies that target the spatiotemporal dynamics and functional transitions of macrophages.

## 1. Introduction

Stroke, clinically termed a cerebrovascular accident (CVA), encompasses two major subtypes: ischemic stroke (IS) and hemorrhagic stroke. Both subtypes induce a spectrum of neurological deficits, leading to significant impairments in patients’ language function, motor control, and other activities of daily living (ADLs) [1]. IS accounts for ~85% of all stroke cases. According to the latest 2025 estimates on stroke from the Global Burden of Disease (GBD) study, there are 93.8 million prevalent cases of stroke and 11.9 million new stroke cases globally [2]. Among noncommunicable diseases (NCDs), stroke remains the second leading cause of death globally (accounting for ~7.0 million annual deaths) and the third leading cause of disability. Furthermore, most stroke patients still need help with daily activities after the onset of the disease. They also face a high risk of stroke recurrence, imposing substantial economic burdens on both their families and society as a whole.

The primary pathological hallmark of IS is cerebral vascular occlusion. This occlusion triggers localized cerebral ischemia and hypoxia, which in turn culminates in neuronal death and subsequent neurological impairment [3]. While intravenous thrombolysis and mechanical thrombectomy are effective in salvaging the ischemic penumbra (the viable but hypoperfused brain tissue surrounding the infarct core). However, their narrow therapeutic time windows and the occurrence of post‐recanalization neurological deterioration highlight that secondary injury, particularly inflammatory injury, plays a pivotal role in determining patients’ overall prognosis. Following IS onset, damage‐associated molecular patterns (DAMPs) released by necrotic neural cells rapidly activate resident microglia, the primary immune effector cells of the central nervous system (CNS). The ensuing neuroinflammatory cascade, orchestrated by activated microglia and peripherally infiltrating macrophages, persists and expands the infarct volume even after reperfusion is achieved. These immune cells migrate across the compromised blood–brain barrier (BBB), exacerbating tissue damage and ultimately leading to irreversible neurological deficits [4]. Single‐cell sequencing evidence indicates that microglia exert a “double‐edged sword” effect throughout the course of IS. In the acute phase, excessive release of pro‐inflammatory cytokines, such as interleukin‐1β (IL‐1β) and tumor necrosis factor‐α (TNF‐α), directly amplifies neuronal injury. In contrast, the delayed activation of anti‐inflammatory signaling pathways in later phases is positively correlated with long‐term neurological functional recovery. This temporal dichotomy highlights that the time window is a critical variable governing the functional transition of microglia [5]. Current clinical treatments for IS, including intravenous thrombolysis, mechanical thrombectomy, and antiplatelet agents, are constrained by three key limitations: narrow therapeutic time windows, inherent bleeding risks, and the high likelihood of thrombosis recurrence. Furthermore, these interventions fail to inhibit the post‐reperfusion inflammatory cascade (a critical driver of secondary brain injury, as noted earlier) and lack the capacity to exert neuroprotective or neuroreparative effects. Thus, to improve clinical outcomes in patients with ischemic brain injury, therapeutic strategies must simultaneously target two key pathological processes: the initial vascular occlusion‐induced damage and the subsequent post‐ischemic inflammatory response [6]. Macrophages exhibit both pro‐inflammatory and reparative functions, and their phenotypic plasticity renders them a promising therapeutic target for IS.

While previous review articles have addressed the role of macrophages in IS, the novelty of this review lies in its spatiotemporal dynamic perspective: it systematically correlates three post‐stroke temporal phases (acute, subacute, and chronic) with three spatial regions (infarct core, penumbra, and remote brain areas), thereby constructing a “spatiotemporal‐functional map” of macrophage behavior.

We focus on analyzing the dynamic changes in the proportions of distinct macrophage subsets, including resident microglia, monocyte‐derived macrophages (MoDMs), and border‐associated macrophages (BAMs) as well as their functional crosstalk across the disease course. Moreover, this review explores emerging regulatory mechanisms (e.g., metabolic‐epigenetic cross‐regulation) in detail, moving beyond the traditional and oversimplified M1/M2 polarization dichotomy. Furthermore, we propose timing‐specific therapeutic strategies tailored to distinct post‐stroke time windows, which provides novel insights for the development of precise macrophage‐targeted immunotherapies for IS.

## 2. Macrophage Subsets and Their Post‐Stroke Dynamics

As a core immune component of the CNS, macrophages serve as a **“signaling hub”**: they perceive cues from the local microenvironment and play a pivotal role in both the maintenance of CNS homeostasis and the promotion of postinjury repair [7]. The major subsets of CNS macrophages include microglia, peripheral MoDMs, and BAMs, each with distinct origins and functional characteristics. Microglia represent the tissue‐resident macrophage population of the CNS and are a key component of the mononuclear phagocyte system; they originate from the embryonic yolk sac [8]. As the CNS‐resident immune sentinels, microglia are indispensable for sustaining CNS steady‐state homeostasis. Their constant surveillance of the neural microenvironment and rapid response to perturbations underpin the maintenance of neural tissue integrity.

In contrast, peripherally derived macrophages (primarily MoDMs) originate from bone marrow‐resident monocytes. During the inflammatory phase of CNS injury or pathology, MoDMs are the dominant infiltrating immune cells, acting as dual regulators that drive both tissue damage (e.g., via excessive pro‐inflammatory cytokine release) and repair processes (e.g., via phagocytosis of cellular debris and secretion of regenerative factors).

BAMs, which localize to meningeal and perivascular spaces of the CNS, exhibit stage‐specific functional roles: they are critically involved in initiating early pro‐inflammatory responses to insults and mediating late‐stage fibrotic remodeling, thereby contributing to the resolution of tissue damage or the progression of pathological fibrosis.

### 2.1. Macrophage Subsets

#### 2.1.1. Microglia

Microglia are the primary resident immune cells of the CNS, with well‐characterized species‐specific developmental trajectories. In mice, microglial progenitors originate from primitive erythromyeloid progenitors (EMPs) within the extraembryonic yolk sac at approximately embryonic day 8.5 (E8.5); these progenitors subsequently migrate into the developing brain via the nascent circulatory system by E9.5, establishing the initial microglial pool [9]. In humans, the spatiotemporal dynamics of microglial development exhibit distinct features: microglial precursors enter the fetal brain around the 4th week of gestation, initiate functional maturation between the 9th and 11th weeks of gestation, and complete colonization of the cerebral cortex after the 17th week of gestation. By approximately the 35th week of gestation, human microglia largely acquire an adult‐like phenotypic profile, laying the foundation for lifelong CNS immune surveillance [10]. Notably, studies have confirmed that microglia maintain a self‐sustaining population through dynamic self‐renewal throughout the lifespan, independent of peripheral monocyte recruitment under physiological conditions [11]. Beyond their canonical immune functions, microglia act as multifunctional regulators of CNS homeostasis: they support the survival, proliferation, and maturation of neurons and neural precursor cells by secreting trophic growth factors (e.g., brain‐derived neurotrophic factor) and clearing apoptotic cellular debris via phagocytosis, thereby directly promoting neurogenesis [12–14]. Furthermore, during both embryonic neural development and adult CNS maintenance, microglia actively participate in the remodeling of synaptic circuits—a process is mediated, at least in part, by the complement C1q/C3 pathway, which tags surplus or dysfunctional synapses for microglial phagocytic elimination [15].

#### 2.1.2. MoDMs

MoDMs differentiate from monocytes, which originate from hematopoietic stem cells (HSCs) in the adult bone marrow; this monocyte pool and by extension, the MoDM population—is continuously replenished throughout the lifespan to maintain functional homeostasis [16]. MoDMs are well recognized for their canonical roles in innate immunity and tissue homeostasis, including the production of pro‐inflammatory mediators, phagocytic clearance of pathogens, and antigen presentation to adaptive immune cells [17]. Conventionally, MoDMs have been categorized based on functional polarization: M1‐type MoDMs primarily drive inflammatory responses by releasing cytotoxic molecules (e.g., reactive oxygen species (ROS), tumor necrosis factor‐α) and inducing target cell death, whereas M2‐type MoDMs facilitate tissue repair processes through phagocytosis of cellular debris and secretion of trophic factors (e.g., transforming growth factor‐β, vascular endothelial growth factor) [18]. It is critical to note that the M1/M2 polarization framework represents a simplified binary model. In the complex in vivo microenvironment—particularly in pathological conditions such as stroke—the actual activation states of MoDMs do not conform to this rigid dichotomy. Instead, MoDMs exhibit a multidimensional and highly plastic functional spectrum, with phenotypes dynamically regulated by local cues (e.g., cytokine gradients, extracellular matrix components). The M1/M2 framework is therefore used herein solely for illustrative purposes; the interpretation of MoDM phenotypes in experimental or clinical contexts should be based on the specific microenvironmental context and integrated analysis of multiomics markers (e.g., transcriptomic, proteomic, and metabolomic profiles).

#### 2.1.3. BAMs

BAMs are strategically localized at the anatomical interfaces between the CNS (i.e., the brain) and the peripheral immune system, including the perivascular spaces (surrounding cerebral blood vessels), choroid plexus (within the ventricular system), and meninges (the protective membranes enveloping the brain). Notably, BAM populations are often enriched in regions adjacent to larger cerebral blood vessels, consistent with their roles in regulating CNS‐periphery crosstalk. Under physiological conditions, BAMs contribute to the maintenance of CNS homeostasis by modulating cerebrospinal fluid (CSF) dynamics (e.g., facilitating CSF circulation and nutrient exchange) and supporting vascular integrity (e.g., preserving vascular elasticity via interactions with vascular smooth muscle cells) [19]. In the pathological context of IS, BAMs exhibit rapid functional remodeling: they actively participate in the recruitment of granulocytes (e.g., neutrophils) to the ischemic lesion and may further exacerbate vascular dysfunction by increasing vascular endothelial permeability—this permeability enhancement can disrupt the BBB and promote the infiltration of peripheral immune cells, thereby amplifying neuroinflammation [19]. Importantly, preclinical studies have demonstrated that targeted reduction of neutrophil infiltration in the acute phase after cerebral ischemia significantly improves neurological outcomes, including reducing infarct volume and enhancing functional recovery [20]. Given their role in early granulocyte recruitment, BAMs are hypothesized to be key upstream mediators of this neutrophil‐driven pathological process.

As a critical “bridge” between the brain parenchyma and the peripheral immune system, BAMs play an indispensable role in orchestrating neuroimmune communication, particularly in translating peripheral inflammatory signals to the CNS and regulating CNS‐derived immune responses. Collectively, these findings indicate that following IS, BAMs contribute to the initiation and amplification of neuroinflammation through two primary mechanisms: 1) recruiting peripheral myeloid cells (e.g., granulocytes and monocytes) to the ischemic region, and 2) releasing pro‐inflammatory mediators (e.g., interleukin‐1β and tumor necrosis factor‐α) that perpetuate local inflammatory cascades [21]. A summary of the major macrophage subsets (including microglia, MoDMs, and BAMs) and their functional roles in IS is provided in Table 1.

**Table 1:** Overview of the major macrophage subsets in ischemic stroke [22–24].

Subset	Origin	Post‐stroke dynamics	Key functions
Microglia	Embryonic yolk sac	M1 polarization in acute phase → M2 shift in subacute phase → Senescence in chronic phase	Synaptic pruning, inflammation initiation, neurotrophic support, and synaptic regulation
MoDMs	Bone marrow Ly6C^hi^ monocytes	Peak infiltration at day 3 → Differentiation into RAMf by day 7	Myelin debris clearance, promotion of angiogenesis
BAMs	Meninges/perivascular spaces	Recruitment of neutrophils → Regulation of BBB permeability	Double‐edged effect: early pro‐inflammation, late fibrotic regulation

### 2.2. Proportional Succession of Macrophage Subsets

Following IS, the three major CNS macrophage subsets, microglia, MoDMs, and BAMs, do not act in isolation. Instead, they engage in a highly coordinated, dynamic spatiotemporal succession process, wherein their functional interactions and phenotypic transitions collectively shape neuroinflammatory progression and ultimately determine tissue repair outcomes [25]. Notably, the macrophage response to stroke exhibits prominent time‐dependent dynamics and spatial heterogeneity: their relative proportions, anatomical distribution, and functional states differ significantly across distinct brain regions, including the infarct core (irreversibly damaged tissue), the peri‐infarct penumbra (viable but at‐risk tissue), and even remote brain regions (areas distant from the infarct but affected by secondary injury cascades) [22].

During the acute phase of stroke (within 72 h post‐ischemia), microglia serve as the first line of defense: they rapidly sense DAMPs and pathogen‐associated molecular patterns (PAMPs) released by injured cells, undergo rapid activation, and become the core drivers of the early neuroinflammatory “storm,” a process that initially restricts tissue damage but may exacerbate secondary injury if unregulated. Subsequently, during the subacute phase (days 3–7 post‐ischemia), peripherally derived MoDMs are rapidly recruited across the disrupted BBB and become the dominant macrophage population in the ischemic region, gradually transitioning from pro‐inflammatory to repair‐promoting phenotypes. By day 5 post‐ischemia, a specialized transitional macrophage subset expressing secreted phosphoprotein 1 (SPP1^+^) emerges; this subset acts as a critical intermediate during the differentiation of pro‐inflammatory MoDMs into repair‐associated macrophages (RAMf). This SPP1^+^‐mediated transitional process lays the molecular and cellular foundation for RAMf to become the primary repair‐mediating force by day 7 post‐ischemia, where they mediate key regenerative processes such as myelin debris clearance, oligodendrocyte precursor cell (OPC) recruitment, and remyelination of damaged axons [22].

**Figure 1:**
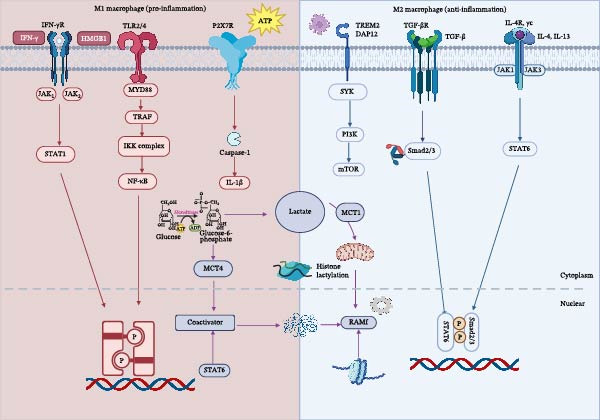
Polarization spectrum and regulatory network of macrophages following ischemic stroke. This schematic integrates the central pathways and crosstalk involving cytokine signaling (e.g., IFN‐γ/STAT1, IL‐4/STAT6), metabolic reprograming (e.g., lactate, glycolysis/OXPHOS), epigenetic modifications (e.g., histone lactylation), and DAMPs in regulating the balance of macrophage M1/M2 polarization.

In the chronic phase of stroke (≥ 14 days post‐ischemia), the CNS microenvironment is characterized by the coexistence of homeostatic restoration and residual pathological risks—a balance tightly regulated by macrophage subset dynamics. On one hand, the relative proportion of microglia within the lesion area increases; these microglia undergo phenotypic refinement to actively participate in reestablishing local tissue homeostasis, primarily through processes such as the clearance of residual cellular debris, the modulation of excessive inflammatory signals, and the support of subtle neural repair [22]. On the other hand, MoDMs, the majority of which are RAMf, still constitute a considerable proportion of the macrophage pool in the chronic phase. While RAMf continue to exert reparative effects (e.g., promoting oligodendrocyte maturation and axonal sprouting), a subpopulation of these cells undergoes phenotypic transition toward a profibrotic state, characterized by the increased secretion of extracellular matrix (ECM) components (e.g., collagen, fibronectin). This profibrotic shift lays the cellular and molecular groundwork for glial scar formation, a process that can restrict aberrant axonal growth while potentially impeding long‐term neural circuit reconstruction [18]. Concurrently, although the relative proportion of BAMs remains relatively stable in the chronic phase, their functional profile undergoes a critical shift: from mediating early, pro‐inflammatory responses (e.g., neutrophil recruitment, pro‐inflammatory cytokine release) during the acute/subacute phases to participating in late‐stage fibrotic regulation (e.g., interacting with astrocytes and fibroblasts to modulate ECM deposition). This stage‐specific functional duality further confirms the “double‐edged sword” nature of BAMs in stroke pathophysiology. They are indispensable for initiating early injury responses and resolving late‐stage tissue damage, yet their dysregulation may contribute to pathological fibrosis [23]. The temporal changes in the relative proportions of these three key macrophage subsets (microglia, MoDMs, and BAMs) within the lesion area post‐stroke, as well as the core functional events associated with each subset at distinct time points, are clearly illustrated in Figure 1 and summarized in Table 2. These multidimensional data provide a quantitative and visual framework for systematically understanding the spatiotemporal dynamics of macrophage subset evolution, which is essential for developing targeted immunomodulatory strategies for stroke therapy.

**Table 2:** Relative proportional changes of major macrophage subsets in the lesion area and key events following ischemic stroke [22].

Time point	Microglia (%)	MoDMs	BAMs (%)	Key events
0–24 h	95	3%	2	Microglial M1 polarization surge [18]
Day 3	35	52% (predominantly Ly6C^hi^)	13	MoDMs infiltration peaks; BAMs recruit neutrophils [22]
Day 5	28	60% (includes SPP1^+^ transitional state)	12	Initiation of reparative RAMf differentiation
Day 7	20	68% (predominantly RAMf)	12	RAMf dominate remyelination [22]
Day 28	45	40% (profibrotic subtype)	12–14	Microglia reestablish homeostasis [26]

*Note*: The proportions in the table are approximate values based on representative study data, intended to illustrate temporal trends. Actual proportions may vary depending on the experimental model, injury severity, and specific brain region.

The numerical succession of macrophage subsets exhibits a highly time‐dependent pattern (Figure 1 and Table 2), accompanied by a functional shift from a pro‐inflammatory (M1) profile in the acute phase to a reparative/homeostatic profile in the subacute and chronic phases. In the acute phase (0–24 h), resident microglia account for over 90% of macrophages in the brain parenchyma. From day 3 onward, peripherally derived MoDMs rapidly infiltrate the brain: their proportion rises to 52%, while the proportion of microglia decreases correspondingly to 35%. By day 7, MoDMs reach their peak proportion of 68%, and microglia decline further to 20%. When entering the chronic phase (day 28), the absolute number of MoDMs decreases, and their proportion drops back to 40%. In contrast, microglia expand via local proliferation, with their proportion rebounding to 45%. BAMs maintain a low baseline abundance throughout the process, gradually increasing from 2% to ~13%–15% [22, 23, 27].

Notably, the reported timelines for macrophage polarization and succession vary across different studies. This heterogeneity likely arises from differences in the animal models used (e.g., permanent vs. transient ischemia), injury severity, detection techniques (e.g., flow cytometry, immunohistochemistry, and single‐cell sequencing), and the specific brain regions examined. This variability underscores the complexity and context‐dependence of the post‐stroke immune response. For example, while some studies report that MoDMs’ infiltration peaks on day 3 [22], others indicate that pro‐inflammatory monocytes remain the dominant population as late as day 5 [28]. The temporal dynamics presented in this review are based on the consensus of most studies. However, it is critical to emphasize that for both practical research and future clinical translation, defining a precise interventional time window requires considering the specific model context and validating findings with multiple detection techniques.

### 2.3. Sex Differences in Macrophages

Accumulating evidence highlights substantial sex‐specific differences in inflammatory responses during secondary brain injury [29], with the quantity, activation status, and functional polarization of macrophages/microglia representing key immunological underpinnings of these disparities. Epidemiological studies demonstrate that while females exhibit a lower incidence of stroke, they experience poorer functional outcomes and higher mortality following stroke onset—particularly as postmenopausal women face a significantly elevated stroke risk [30]. Elucidating the cellular and molecular mechanisms driving these sex‐related differences will yield critical evidence to support sex‐stratified strategies in precision immunotherapy for stroke.

Existing evidence indicates that this sexual dimorphism is particularly prominent in macrophag‐mediated neuroinflammatory responses, although its cytological basis remains incompletely defined. At the cellular level, microglia—the resident macrophages of the CNS—exhibit striking sex‐specific differences following ischemic injury. Studies have demonstrated that microglia in young male mice preferentially polarize toward pro‐inflammatory phenotypes after ischemic insult, accompanied by elevated secretion of pro‐inflammatory cytokines. In contrast, microglia in young female mice more readily adopt a reparative phenotype, characterized by enhanced clearance of cellular debris and improved tissue repair. In addition, the recruitment and polarization of peripheral monocytes also display sex‐dependent distinctions. Research has shown that macrophages in female mice adopt an anti‐inflammatory profile following stroke, with higher CD206 expression relative to male mice, indicative of a greater tendency toward reparative phenotypic switching [31].

At the hormonal regulatory level, sex hormones serve as key mediators of these sex‐specific differences. Estrogen directly modulates macrophage function via estrogen receptor α (ERα), inhibiting disease‐associated microglia (DAM) gene expression and suppressing glycolytic metabolic reprograming (mTOR) [32], thereby constraining excessive neuroinflammatory responses. Conversely, ERα deficiency contributes to microglial dysfunction and amplified pro‐inflammatory reactions following ischemic injury [29]. Progesterone exerts neuroprotective effects by promoting M2 polarization and inhibiting the NLRP3 inflammasome (attenuating IL‐1βmaturation) [33]. In contrast, androgens regulate macrophage polarization through modulation of Toll‐like receptor 4 (TLR4) expression: macrophages from male individuals exhibit higher TLR4 levels, leading to increased production of chemokines such as CXCL10 and subsequent exacerbation of inflammatory responses [34]. Collectively, these findings highlight the necessity of fully integrating sex‐related factors into future macrophage‐targeted immunomodulatory therapies to achieve more precise individualized interventions.

At the metabolic level, microglial mTOR exhibits substantial sex‐specific differences. During the acute phase of IS, male microglia display severe mitochondrial dysfunction and struggle to meet energy metabolic requirements, whereas female microglia maintain robust oxidative phosphorylation (OXPHOS) capacity to support phagocytic functions [32]. However, this metabolic pattern reverses with aging: in senescent individuals, hippocampal microglia from females exhibit enhanced glycolytic switching via the AKT‐mTOR‐HIF‐1α pathway [35], accompanied by a significantly higher abundance of DAM—particularly the DAM2 subpopulation—compared to aged males. Notably, estrogen supplementation can downregulate DAM gene expression and glycolytic activity, suggesting that hormone replacement therapy may restore metabolic homeostasis in aged female microglia [32].

## 3. Lineage Polarization and Regulatory Mechanisms of Macrophages

**Figure 2:**
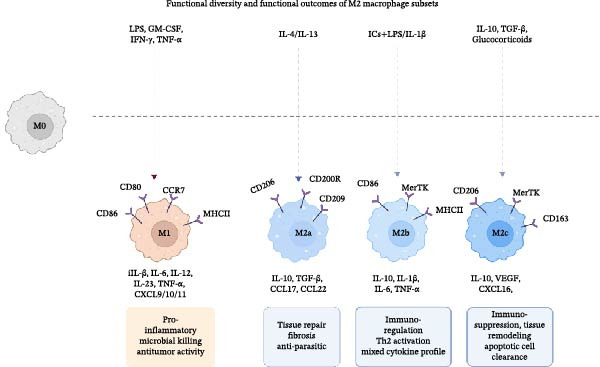
Functional diversity and functional outcomes of M2 macrophage subsets. M2 macrophages are further subdivided into M2a, M2b, and M2c subsets on the basis of stimulating cues and effector functions. M2a cells (elicited by IL‐4/IL‐13) drive Th2 activation, fibrosis, and antiparasitic responses; M2b cells (triggered by immune complexes plus TLR agonists) display a mixed pro‐ and anti‐inflammatory cytokine profile and participate in immunoregulation; M2c cells (induced by IL‐10, TGF‐β, or apoptotic cells) are specialized in immunosuppression, tissue repair, apoptotic‐cell clearance, and antitumor activity. Together, these subsets illustrate the functional plasticity of the M2 phenotype in post‐stroke recovery and other pathological contexts.

Macrophage polarization refers to a plastic process: in response to specific microenvironmental signals, macrophages become activated at precise spatiotemporal nodes and thereby acquire distinct functional phenotypes [36]. A consensus has now been reached in the field regarding how to describe the broad categories of macrophage activation states. These phenotypic shifts are mainly driven by cytokines and are further regulated by metabolites (Figure 2).

### 3.1. Spectrum of Macrophage Polarization

The M1/M2 framework is widely used to describe macrophage activation states, and these phenotypic changes are primarily driven by cytokines. For example, M1 macrophages are induced in an inflammatory microenvironment dominated by Toll‐like receptor (TLR) signaling and interferon signaling. By contrast, M2 macrophages are functionally heterogeneous and typically arise in environments dominated by Th2‐type immune responses [37]. This group can be further divided into several subtypes: M2a, M2b, and M2c (Figure 2). The M2a subtype, induced by IL‐4 and IL‐13, promotes fibrosis and supports Th2‐type immunity. The M2b subtype, activated by immune complexes in combination with TLR agonists, secretes high levels of IL‐10 and exerts potent immunoregulatory effects. The M2c subtype, induced by IL‐10, TGF‐β, or apoptotic cells, exhibits strong anti‐inflammatory activity, mediates apoptotic‐cell clearance, and contributes to inflammation resolution and tissue remodeling [38].

### 3.2. Regulatory Network

Following IS, the brain microenvironment undergoes profound and dynamic remodeling. This remodeling drives the transition of macrophages from an M1‐like to an M2‐like phenotype, representing a core immune mechanism underlying self‐repair. This phenotypic transition is primarily regulated by three microenvironmental cues: cytokines, metabolic signals, and DAMPs.

#### 3.2.1. Cytokine Network Switching

In IS, the activation of macrophages undergoes a phenotypic transition: from being dominated by pro‐inflammatory factors (e.g., IFN‐γ, TNF‐α) in the early phase to being dominated by anti‐inflammatory repair factors in later stages, including IL‐4/IL‐13 (which acts via the STAT6 pathway) and IL‐10 and TGF‐β (which act via the Smad pathway) [39–41].

IFN‐γ is a key cytokine inducing M1 polarization, primarily produced by activated Th1 cells, among others. Granulocyte‐macrophage colony‐stimulating factor (GM‐CSF) is another important cytokine, as it promotes macrophage polarization toward an M1‐like phenotype. Macrophages stimulated by GM‐CSF show enhanced antigen presentation, complement‐ and antibody‐mediated phagocytosis, bactericidal activity, and leukocyte chemotaxis and adhesion [39]. These M1‐like macrophages serve as key effectors in amplifying the inflammatory response during the acute phase.

In contrast to M1‐polarizing factors, M2 polarization is primarily driven by cytokines such as IL‐4, IL‐13, IL‐10, and TGF‐β. The signaling pathways and functions of these cytokines differ across the various stages of IS. The IL‐4/STAT6 pathway acts through a three‐step “receptor‐kinase‐transcription factor” cascade to induce the M2a phenotype and drive the expression of anti‐inflammatory and reparative genes. This pathway provides a molecular framework for the transition from acute inflammation to reparative immunity during stroke recovery [42]. The TGF‐β signaling pathway, primarily via Smad2/3, is a core driver of macrophage polarization toward the M2c phenotype. M2c macrophages exert immunoregulatory and tissue‐remodeling functions, offering both a theoretical basis and potential therapeutic targets for inflammation resolution and tissue regeneration after stroke [43, 44]. Additionally, the TREM2‐DAP12‐SYK signaling axis, upon recognizing apoptotic cells, activates downstream PI3K/ERK pathways and lipid mTOR (e.g., via mTOR). This forms a positive feedback loop that significantly enhances macrophage efferocytosis and the sustained release of reparative factors, making this axis a crucial molecular hub for the transition from inflammation resolution to tissue regeneration after stroke [45–47].

#### 3.2.2. mTOR

Lactate accumulation directly promotes the expression of M2‐like phenotype‐associated genes through epigenetic modifications such as histone lactylation (e.g., H3K18la) [48]. This phenotypic switch is biologically critical: the early M1‐like response clears necrotic debris and defends against infection, while a timely shift to the M2‐like phenotype terminates destructive inflammation and limits secondary brain injury. M2‐like macrophages clear apoptotic debris via efferocytosis, thereby removing obstacles to tissue regeneration. They also actively secrete trophic factors (e.g., IGF‐1, VEGF, GDNF), which directly promote angiogenesis, remyelination, and synaptic remodeling. Thus, a deeper understanding of the mechanisms by which the brain microenvironment drives this phenotypic switch and strategies to promote its timely and region‐specific occurrence represents a promising direction for developing IS immunotherapies.

**Figure 3:**
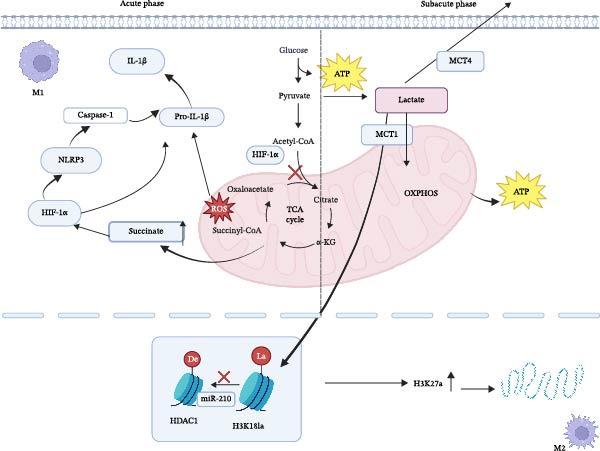
Glucose‐derived lactate is exported via MCT4 (M1) or imported through MCT1 (M2). Extracellular lactate inhibits HDAC1 via the HIF‐1α/miR‐210 axis, elevating H3K18la and promoting M2‐related gene expression while restraining NLRP3–caspase‐1–dependent IL‐1β release. TCA‐cycle intermediates and ROS generated during OXPHOS further reinforce this metabolic‐epigenetic switch.

Recent studies have revealed that lactate accumulation in the ischemic region suppresses HDAC1‐3 via the HIF‐1α/miR‐210 axis, leading to a simultaneous increase in both H3K27ac and H3K18la modifications. These modifications synergistically accelerate M2‐like polarization [49] (Figure 3). This mechanism acts in concert with histone lactylation (e.g., H3K18la) to remodel chromatin accessibility, thereby activating reparative pathways such as the IL‐4/STAT6 pathway [50]. Furthermore, the lactate transporter MCT1 mediates lactate influx into mitochondria, which promotes OXPHOS and the M2‐like phenotype. In contrast, the transporter MCT4 maintains high glycolytic flux by exporting lactate, thereby reinforcing the pro‐inflammatory M1‐like phenotype [51]. This coupling between mTOR and epigenetic modification provides a spatiotemporally precise, dual regulatory mechanism for the transition of macrophages toward a reparative phenotype (Figure 3).

#### 3.2.3. Evolution of DAMPs

During the early phase of IS, DAMPs such as HMGB1 and ATP promote M1‐like macrophage polarization. Subsequently, apoptotic cells trigger efferocytosis via receptors including TREM2, thereby inducing a reparative M2‐like phenotype [52]. After the TREM2‐driven M2‐like phenotype is established, late‐stage DAMPs derived from apoptotic cells do not stop signaling; instead, they continue to provide “fuel” for the metabolic and epigenetic reprograming essential for tissue repair [22]. This shift in the DAMP profile—from early alarmins (e.g., HMGB1, ATP) to late‐stage apoptosis‐associated molecular patterns (e.g., phosphatidylserine)—serves as a critical upstream signal that orchestrates the temporal shift of macrophages from the M1 to the M2 phenotype [53, 54].

## 4. Spatiotemporal Dynamics of Macrophages in the Pathophysiology of IS

As previously mentioned, macrophage subsets undergo complex proportional succession and phenotypic polarization shifts following IS. These spatiotemporal dynamics fundamentally determine their functional roles throughout the entire course of brain injury. In the following sections, we systematically examine the central roles and regulatory mechanisms of macrophages in key pathophysiological processes, including neuroinflammation, tissue repair, and fibrosis across the acute, subacute, and chronic phases of stroke.

### 4.1. Acute Phase

During the acute phase of IS (spanning minutes to days postinsult), activated microglia and infiltrating MoDMs serve as the primary immune effectors driving secondary brain injury. Their rapid activation and infiltration constitute the core of the neuroinflammatory response.

Following ischemic brain injury, microglia sense extracellular ATP via the P2Y12 receptor, which directs their migration toward the lesion site [55]. They subsequently polarize toward a pro‐inflammatory M1‐like phenotype. Upon NF‐κB pathway activation, they persistently secrete large quantities of pro‐inflammatory cytokines (e.g., TNF‐α, IL‐1β, IL‐6), chemokines, and ROS [56]. This pro‐inflammatory response directly exacerbates neuronal death and neuroinflammation, induces the death of oligodendrocytes and OPCs, and inhibits remyelination and neural precursor cell proliferation, collectively contributing to secondary brain injury [18].

Furthermore, macrophages derived from pro‐inflammatory Ly6*C* ^+^ monocytes extensively infiltrate the lesion [57], intensifying the inflammatory response and contributing to BBB disruption. This process expands the infarct core, induces neuronal apoptosis or necrosis, suppresses neurogenesis, and impedes early tissue repair. Within 3 h of reperfusion, the NLRP3 inflammasome begins to assemble. IL‐1β secretion peaks at 12 h and remains elevated for up to 72 h, marking a sustained inflammatory phase, which further amplifies the inflammatory cascade [58]. Thereby further amplifying the inflammatory cascade.

During the acute phase of IS, macrophages drive the neuroinflammatory cascade via a “DAMPs‐PRRs signaling storm.“ Within minutes of stroke onset, high‐mobility group box 1 (HMGB1) and extracellular ATP released from necrotic neurons activate TLR4 and P2X7 receptors on microglia, respectively. This triggers the nuclear factor‐κB (NF‐κB) and NLRP3 inflammasome pathways, leading to a burst of pro‐inflammatory cytokines [5]. Concurrently, mitochondrial DNA (mtDNA) released from damaged mitochondria enhances type I interferon responses via the cGAS‐STING axis, creating a synergistic amplification effect with TLR4 signaling [59]. This process is accompanied by a glycolytic burst that enhances IL‐1β production and succinate accumulation, both of which contribute to the sustained amplification of inflammation [60]. Furthermore, within 24 h, peripherally infiltrated monocytes become the dominant population at the inflammatory focus, establishing a positive feedback loop with microglia. This interaction further activates microglia, driving their polarization toward the M1‐like phenotype and enabling them to directly induce neuronal apoptosis via granzyme B [61].

### 4.2. Subacute Phase

Approximately 1 week after an IS, microglia undergo a critical phenotypic transition from a pro‐inflammatory to a reparative state. Meanwhile, RAMf, derived from MoDMs, emerge as key regulators of inflammation resolution and tissue repair. During this subacute phase, peripherally derived Ly6*C* ^+^CCR2^+^ MoDMs (Ly6*C* ^+^CCR2^+^ MoDMs) become the predominant macrophage population. They extensively infiltrate the infarct core by post‐stroke day 3. At this stage, M1‐like microglia continue to exert a key pro‐inflammatory role, while the number of M2‐like microglia decreases, accompanied by a significant shift in population composition: Ly6*C* ^+^CCR2^+^ MoDMs account for 52% of total macrophages, microglia for 35%, and BAMs for 13%. By post‐stroke day 7, the proportion of RAMf increases markedly to 68%, establishing them as the dominant macrophage subset [18, 62].

RAMf promote remyelination by clearing myelin debris and secreting trophic and pro‐remyelinating factors [63, 64]. Remyelination is a natural protective and regenerative response to IS, and its efficacy depends on a favorable microenvironment—characterized by reduced myelin debris, sufficient lipid availability, and anti‐inflammatory signaling. Furthermore, RAMf contribute to capillary proliferation by secreting factors such as IGF‐1, VEGF‐B, and PDGF‐Rα. Their sustained phagocytic function, which is partly regulated by the transcription factor Mafb, supports their role in facilitating both remyelination and angiogenesis after IS [64]. The subacute phase serves as a critical window for macrophage phenotypic commitment; once established, the RAMf identity can be maintained epigenetically into the chronic phase.

### 4.3. Chronic Phase

The chronic phase typically spans from post‐stroke day 14 to 3–6 months or beyond. During this period, a small yet persistent population of M1‐like macrophages sustains chronic low‐grade inflammation by secreting factors such as CXCL10 and IL‐1β. This persistent inflammation may recruit autoreactive T cells, potentially contributing to secondary neurodegeneration in remote brain regions such as the thalamus [65]. Concurrently, a larger population of M2‐like macrophages, predominantly RAMf, persists. Their functional state reflects a dynamic balance among tissue repair, profibrotic activity, and senescence. These long‐residing RAMf continue to support remyelination, rebuild the neurovascular unit, promote synaptic remodeling, and sustain efferocytosis to clear dead cells and apoptotic myelin debris, thereby preventing secondary inflammation. However, when RAMf migrate to remote regions, their excessively activated phagocytic activity may conversely induce secondary neuronal degeneration [66]. More importantly, within the lesion core, a subset of RAMf undergoes transformation into myofibroblast‐like macrophages. Driven by the CD9‐TGF‐β/Smad3 axis, these transformed cells actively participate in glial scar formation [67]. Studies have shown that targeted inhibition of CD9 or TGF‐β receptor 1 (TGFβR1) can suppress this profibrotic transition while preserving the reparative functions of RAMf [26].

**Figure 4:**
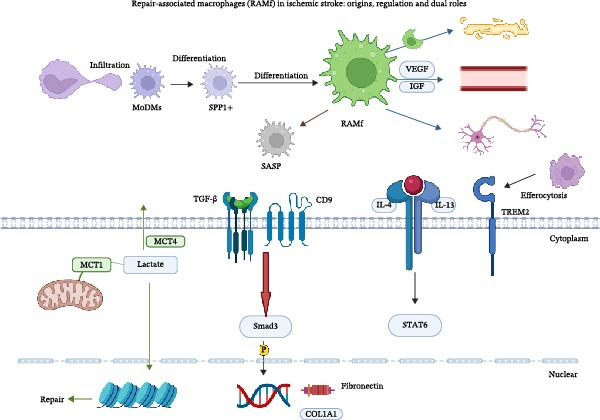
Metabolic and signaling signature of repair‐associated macrophages (RAMf) after stroke. RAMf up‐regulates MCT1 (but not MCT4) to import lactate, which enhances STAT6 and Smad3 activation via histone lactylation (H3K18la) and IL‐4/IL‐13 signaling, while secreting VEGF and IGF to promote angiogenesis and remyelination. Concurrent COL1A1 expression driven by TGF‐β/Smad3 underlies fibrotic scar formation, illustrating the dual repair/fibrosis role of RAMf.

However, during the chronic phase of IS, brain‐resident macrophages acquire a senescence‐like phenotype, characterized by cell cycle arrest [68], mitochondrial dysfunction, and mTOR [69]. This phenotype not only hinders neurological repair but may also drive secondary degeneration in remote brain regions via the senescence‐associated secretory phenotype (SASP). Here, we summarize the primary roles and key regulatory targets of macrophages across these three phases in Table 3 and Figure 4.

**Table 3:** Main roles and key regulatory targets of macrophages in three periods [5, 26, 64, 65].

Phase	Neurotoxic effects	Reparative roles	Key regulatory
Acute phase	• Release ROS/TNF‐α → neuronal death • Disrupt BBB integrity	• Initiate debris clearance • Contain lesion expansion	HMGB1–TLR4 NLRP3 inflammasome ATP–P2X7R
Subacute phase	Persistent inflammation → secondary injury	• Clear myelin debris • Secrete IGF‐1/VEGF → angiogenesis	TREM2, PPARγ/STAT6 Mafb
Chronic phase	• CXCL10 release → T‐cell recruitment • Fibrotic transition → glial scar	• Promote synaptic remodeling • Efferocytosis limits inflammation	CD9‐TGFβR, Senescence‐related pathways (e.g., SASP)

## 5. Therapeutic Strategies Targeting Macrophages

Following IS, macrophages are not only key mediators of the inflammatory response but also play pivotal roles in tissue repair, inflammation resolution, and neuroregeneration. Therefore, developing targeted strategies to modulate macrophage activity at different stages of stroke pathogenesis holds significant therapeutic potential.

### 5.1. Acute Phase

During the acute phase (0–72 h) of IS, the primary focus is to suppress the neuroinflammatory cascade. Key therapeutic strategies include (1) targeted depletion of BAMs within 24–72 h. Rodent proof‐of‐concept studies have validated this approach, though clinical translation will require the development of novel brain‐penetrant CSF‐1R inhibitors to block BAMs’ role in recruiting neutrophils and exacerbating BBB disruption [21, 70, 71]; (2) blockade of early DAMP signaling, such as using anti‐HMGB1 antibodies or P2X7 receptor antagonists, to prevent initial microglial activation [72, 73].

### 5.2. Subacute Phase

During the subacute phase (3–7 days), therapeutic focus shifts toward promoting the phenotypic transition of macrophages toward a reparative state. Key therapeutic strategies include: (1) exogenously administering cytokines such as IL‐4 and IL‐13 to activate the STAT6 pathway, thereby driving macrophages toward an M2a‐like reparative phenotype [74]; (2) administering TREM2 agonists to enhance macrophage efferocytosis, clear apoptotic debris, and lay the foundation for tissue repair [75]; and (3) using M2 macrophage membrane‐coated biomimetic nanoparticles (M2‐NPs) for targeted delivery of anti‐inflammatory factors, which efficiently induces the local brain microenvironment to shift toward a reparative state [76].

### 5.3. Chronic Phase

The therapeutic goal of the chronic phase (≥14 days) of IS is to maintain brain homeostasis and counteract detrimental phenotypic transformations of macrophages. Key therapeutic strategies include (1) TGFβRⅠ inhibitors or anti‐CD9 antibodies to suppress the transition of RAMf into a profibrotic phenotype and reduce glial scar formation [77, 78]; (2) senolytic agents to eliminate senescent macrophages, thereby reducing SASP‐mediated chronic inflammation and secondary neurodegeneration [68].

These strategies hold significant promise for modulating the post‐stroke immune microenvironment via macrophage‐targeted interventions. However, future research needs to focus on addressing the impact of gender differences on macrophage response, identifying the optimal time window for intervention, and developing novel delivery systems that can efficiently cross the BBB and target specific macrophage subsets to promote the clinical translation of these strategies.

## 6. Limitations in Translating Mouse Macrophage Kinetics to Humans

It is explicitly emphasized that the spatiotemporal dynamics and phenotypic transition mechanisms of macrophages addressed in this review are predominantly derived from experimental investigations employing mouse models of IS. While these models constitute an essential basis for deciphering neuroimmune mechanisms, fundamental interspecies differences between mice and humans considerably restrict the direct clinical translation of the corresponding research findings.

First, there are significant differences in the time course of macrophage‐mediated inflammatory responses between mice and humans. In mouse IS models, MoDMs typically reach peak infiltration at day 3 post‐ischemia and maximum accumulation at day 7, with RAMf becoming the dominant population within the first week. In contrast, macrophage infiltration progresses more slowly in human stroke. Anttila et al. [79], through direct comparison of MANF protein expression kinetics in phagocytic microglia/macrophages between human and rat stroke, found that microglia/macrophage numbers in ischemic brain tissue peaked at ~2 weeks post‐stroke in patients [79], whereas in rats, the peak occurred at day 7 [80]. This significant delay in the time course suggests that the acute‐to‐subacute transition period, which constitutes a critical therapeutic window in rodents, may be significantly delayed and prolonged in human patients, thereby potentially directly affecting the timing window for immunomodulatory interventions.

Second, macrophage markers and phenotypic definitions exhibit substantial divergence across species. Classical murine markers, including Ly6C, F4/80, and CD206, do not fully align with human monocyte/macrophage subpopulations. Such inconsistencies in marker expression impede direct cross‐species comparisons of polarization states and complicate the functional interpretation of corresponding findings [81].

Third, conventional mouse models of stroke possess inherent limitations. Such models predominantly employ young, genetically homogeneous animals (e.g., 8–12‐week‐old C57BL/6 mice) to generate standardized, controllable cortical infarct lesions. In contrast, human stroke patients display pronounced heterogeneity, including advanced age and frequent comorbidities such as hypertension, diabetes mellitus, and atherosclerosis, along with substantial variability in infarct location and volume. These clinical comorbidities and phenotypic differences are difficult to fully recapitulate in standard preclinical animal models [82].

Fourth and finally, marked interspecies differences also exist during the long‐term post‐stroke repair phase. Tissue remodeling in rodents is generally largely accomplished within weeks [83], whereas macrophages in human patients remain engaged in phagocytosis and inflammatory modulation for months [84], indicating potential temporal discrepancies in repair‐phase therapeutic strategies across species. These species‐specific disparities exert direct and profound impacts on the clinical translation of macrophage‐targeted interventions. Collectively, these observations underscore that extreme caution is required when advancing experimental model–derived macrophage‐based therapies toward clinical application. Priority should be placed on validating findings from mouse studies in human stroke specimens and developing translational models that more faithfully recapitulate human neuroimmune responses.

## 7. Conclusions and Perspectives

Macrophages are central players in the post‐stroke immune response, and their dynamic polarization profoundly influences the balance between neural injury and repair. Understanding their spatiotemporal heterogeneity and underlying regulatory networks is crucial for developing novel therapeutic strategies. This review systematically elaborates on the dual and context‐dependent role of macrophages in IS. In the acute phase, macrophages drive a destructive neuroinflammatory storm via the DAMPs/PRRs/inflammasome axis (e.g., HMGB1/TLR4, ATP/P2X7R). In the subacute phase, they transition to a reparative phenotype exemplified by RAMf through metabolic–epigenetic reprograming, thereby promoting inflammation resolution and tissue repair processes such as remyelination and angiogenesis. Notably, during the chronic phase, macrophages, particularly RAMf, exhibit a complex functional balance among repair, fibrosis, and senescence. Their transition toward a profibrotic phenotype significantly impedes full recovery but also represents a potential therapeutic target for antifibrotic intervention. Additionally, the senescent phenotype of macrophages—potentially driven by the SASP—may exacerbate long‐term neural damage.

Future research should leverage single‐cell and spatial multiomics technologies to resolve the spatial localization and interaction networks of macrophage subsets with higher resolution. A key challenge is to elucidate how mTOR (e.g., driven by lactate and succinate) precisely couples with epigenetic modifications (e.g., lactylation and acetylation) to spatiotemporally “encode” macrophage fate. Ultimately, targeting these metabolic–epigenetic checkpoints may shift the therapeutic paradigm from passive suppression of inflammation to active reprograming of the immune microenvironment, ushering in a new era of neural repair for IS. The full literature search strategy, including database‐specific query strings and keyword combinations, is provided in the Supporting Information.

## Funding

This study was provided by grants from the Department of Education of Jilin Province (Grant JJKH20250164KJ) and the Graduate Innovation Fund of Jilin University (Grant 2025CX280).

## Conflicts of Interest

The authors declare no conflicts of interest.

## Supporting Information

Additional supporting information can be found online in the Supporting Information section.

## Supporting information


**Supporting Information** Method This review retrieved literature published from January 2000 to December 2025 by searching the PubMed, Web of Science and Embase databases. The search keywords included: “macrophage”, “microglia”, “stroke”, “cerebral ischemia”, “inflammation”, “neuroinflammation”, “M1/M2 polarization”. Inclusion criteria: (1) Peer‐reviewed English journal articles; (2) In vivo or in vitro studies related to the functions of post‐stroke macrophages and microglia; (3) Studies providing mechanistic insights into macrophage polarization, spatiotemporal dynamics, or functional roles. Exclusion criteria: (1) Abstracts of conferences, editorials and reviews; (2) Non‐English literature; (3) Literature whose full text could not be obtained; (4) Studies focusing exclusively on hemorrhagic stroke without ischemic components. Finally, all included literature was qualitatively and comprehensively analyzed based on thematic relevance.

## Data Availability

The data that support the findings of this study are available upon request from the corresponding author. The data are not publicly available due to privacy or ethical restrictions.

## References

[1] McBride K. L. , White C. L. , and Sourial R. , et al.Postdischarge Nursing Interventions for Stroke Survivors and Their Families, Journal of Advanced Nursing. (2004) 47, no. 2, 192–200, 10.1111/j.1365-2648.2004.03078.x, 2-s2.0-3242662503. 15196193

[2] Feigin V. L. , Brainin M. , and Norrving B. , et al.World Stroke Organization: Global Stroke Fact Sheet 2025, International Journal of Stroke. (2025) 20, no. 2, 132–144, 10.1177/17474930241308142. 39635884 PMC11786524

[3] Salaudeen M. A.-O. , Bello N. , and Danraka R. N. , et al.Understanding the Pathophysiology of Ischemic Stroke: The Basis of Current Therapies and Opportunity for New Ones, Biomolecules. (2024) 8. 10.3390/biom14030305 PMC10968326 38540725

[4] Takeshita Y. and Ransohoff R. M. , Inflammatory Cell Trafficking across the Blood-Brain Barrier: Chemokine Regulation and In Vitro Models, Immunological Reviews. (2012) 248, no. 1, 228–239, 10.1111/j.1600-065X.2012.01127.x, 2-s2.0-84862741839. 22725965 PMC3383666

[5] Cheng C. Y. and Lee Y. C. , Anti-Inflammatory Effects of Traditional Chinese Medicines against Ischemic Injury in In Vivo Models of Cerebral Ischemia, Evidence-Based Complementary and Alternative Medicine. (2016) 2016, no. 1, 10.1155/2016/5739434, 2-s2.0-84989337962, 5739434. 27703487 PMC5040804

[6] Jean W. C. , Spellman S. R. , and Nussbaum E. S. , et al.Reperfusion Injury After Focal Cerebral Ischemia: The Role of Inflammation and the Therapeutic Horizon, Neurosurgery. (1998) 43, no. 6, 1382–1396, 10.1227/00006123-199812000-00076. 9848853

[7] Fang Y. P. , Qin Z. H. , and Zhang Y. , et al.Implications of Microglial Heterogeneity in Spinal Cord Injury Progression and Therapy, Experimental Neurology. (2023) 359, 10.1016/j.expneurol.2022.114239, 114239. 36216123

[8] Prinz M. and Priller J. , Microglia and Brain Macrophages in the Molecular Age: From Origin to Neuropsychiatric Disease, Nature Reviews: Neuroscience. (2014) 15, no. 5, 300–312, 10.1038/nrn3722, 2-s2.0-84899418705. 24713688

[9] Ginhoux F. , Greter M. , and Leboeuf M. , et al.Fate Mapping Analysis Reveals that Adult Microglia Derive From Primitive Macrophages, Science. (2010) 330, no. 6005, 841–845, 10.1126/science.1194637, 2-s2.0-78149360132. 20966214 PMC3719181

[10] Menassa D. A. , Muntslag T. A. O. , and Martin-Estebané M. , et al.The Spatiotemporal Dynamics of Microglia Across the Human Lifespan, Developmental Cell. (2022) 57, no. 17, 2127–2139.e6, 10.1016/j.devcel.2022.07.015. 35977545 PMC9616795

[11] Askew K. , Li K. , and Olmos-Alonso A. , et al.Coupled Proliferation and Apoptosis Maintain the Rapid Turnover of Microglia in the Adult Brain, Cell Reports. (2017) 18, no. 2, 391–405, 10.1016/j.celrep.2016.12.041, 2-s2.0-85009165788. 28076784 PMC5263237

[12] Eyo U. B. , Mo M. , and Yi M. H. , et al.P2Y12R-Dependent Translocation Mechanisms Gate the Changing Microglial Landscape, Cell Reports. (2018) 23, no. 4, 959–966, 10.1016/j.celrep.2018.04.001, 2-s2.0-85045917302. 29694903 PMC5965271

[13] Nimmerjahn A. , Kirchhoff F. , and Helmchen F. , Resting Microglial Cells Are Highly Dynamic Surveillants of Brain Parenchyma In Vivo, Science. (2005) 308, no. 5726, 1314–1318, 10.1126/science.1110647, 2-s2.0-19744380563. 15831717

[14] Aarum J. , Sandberg K. , and Haeberlein S. L. , et al.Migration and Dfferentiation of Neural Precursor Cells Can be Directed by Microglia, *Proceedings of the National Academy of Sciences of the United States of America*, 2003, 15983–15988. 10.1073/pnas.2237050100 PMC307679 14668448

[15] Schafer D. P. , Lehrman E. K. , and Kautzman A. G. , et al.Microglia Sculpt Postnatal Neural Circuits in an Activity and Complement-Dependent Manner, Neuron. (2012) 74, no. 4, 691–705, 10.1016/j.neuron.2012.03.026, 2-s2.0-84861427387. 22632727 PMC3528177

[16] Schulz C. , Perdiguero E. G. , and Chorro L. , et al.A Lineage of Myeloid Cells Independent of Myb and Hematopoietic Stem Cells, Science. (2012) 336, no. 6077, 86–90, 10.1126/science.1219179, 2-s2.0-84859508307. 22442384

[17] Mosser D. M. , Hamidzadeh K. , and Goncalves R. , Macrophages and the Maintenance of Homeostasis, Cellular & Molecular Immunology. (2021) 18, no. 3, 579–587, 10.1038/s41423-020-00541-3. 32934339 PMC7491045

[18] Wicks E. E. , Ran K. R. , and Kim J. E. , et al.The Translational Potential of Microglia and Monocyte-Derived Macrophages in Ischemic Stroke, Frontiers in Immunology. (2022) 13, 10.3389/fimmu.2022.897022, 897022. 35795678 PMC9251541

[19] Drieu A. , Du S. , and Storck S. E. , et al.Parenchymal Border Macrophages Regulate the Flow Dynamics of the Cerebrospinal Fluid, Nature. (2022) 611, no. 7936, 585–593, 10.1038/s41586-022-05397-3. 36352225 PMC9899827

[20] Dhanesha N. , Patel R. B. , and Doddapattar P. , et al.PKM2 Promotes Neutrophil Activation and Cerebral Thromboinflammation: Therapeutic Implications for Ischemic Stroke, Blood. (2022) 139, no. 8, 1234–1245, 10.1182/blood.2021012322. 34529778 PMC8874361

[21] Yu N. , Zhao Y. , and Wang P. , et al.Changes in Border-Associated Macrophages After Stroke: Single-Cell Sequencing Analysis, Neural Regeneration Research. (2026) 21, no. 1, 346–356, 10.4103/NRR.NRR-D-24-01092. 39927762 PMC12094533

[22] Zhang X. , Li H. , and Gu Y. , et al.Repair-Associated Macrophages Increase After Early-Phase Microglia Attenuation to Promote Ischemic Stroke Recovery, Nature Communications. (2025) 16, no. 1, 10.1038/s41467-025-58254-y, 3089. PMC11958652 40164598

[23] Dias D. O. , Kalkitsas J. , and Kelahmetoglu Y. , et al.Pericyte-Derived Fibrotic Scarring Is Conserved Across Diverse Central Nervous System Lesions, Nature Communications. (2021) 12, no. 1, 10.1038/s41467-021-25585-5, 5501. PMC8448846 34535655

[24] Kanazawa M. , Ninomiya I. , and Hatakeyama M. , et al.Microglia and Monocytes/Macrophages Polarization Reveal Novel Therapeutic Mechanism Against Stroke, International Journal of Molecular Sciences. (2017) 18, no. 10, 10.3390/ijms18102135, 2-s2.0-85032855440, 2135. 29027964 PMC5666817

[25] Shui X. , Chen J. , and Fu Z. , et al.Microglia in Ischemic Stroke: Pathogenesis Insights and Therapeutic Challenges, Journal of Inflammation Research. (2024) 17, 3335–3352, 10.2147/JIR.S461795. 38800598 PMC11128258

[26] Huang W. , Zhang Z. , and Li X. , et al.CD9 Promotes TβR2-TβR1 Association Driving the Transition of Human Dermal Fibroblasts to Myofibroblast Under Hypoxia, Molecular Medicine. (2024) 30, no. 1, 2024–2162, 10.1186/s10020-024-00925-5. PMC11428569 39333849

[27] Wang Y. , Liu W. , and Geng P. , et al.Role of Crosstalk between Glial Cells and Immune Cells in Blood-Brain Barrier Damage and Protection After Acute Ischemic Stroke, Aging and Disease. (2023) 15, 2507–2525, 10.14336/AD.2023.1010. 37962453 PMC11567273

[28] Fang W. , Zhai X. , and Han D. , et al.CCR2-Dependent Monocytes/Macrophages Exacerbate Acute Brain Injury but Promote Functional Recovery After Ischemic Stroke in Mice, Theranostics. (2018) 8, no. 13, 3530–3543, 10.7150/thno.24475, 2-s2.0-85048339363. 30026864 PMC6037034

[29] Cordeau P. , Hébert M. , Weng Y. C. , and Kriz J. , Estrogen Receptors Alpha Mediates Postischemic Inflammation in Chronically Estrogen-Deprived Mice, Neurobiology of Aging. (2016) 40, 50–60, 10.1016/j.neurobiolaging.2016.01.002, 2-s2.0-84960408092. 26973103

[30] Kerr N. , Dietrich D. W. , and Bramlett H. M. , et al.Sexually Dimorphic Microglia and Ischemic Stroke, CNS Neuroscience & Therapeutics. (2019) 25, no. 12, 1308–1317, 10.1111/cns.13267. 31747126 PMC6887716

[31] Seifert H. A. , Benedek G. , and Liang J. , et al.Sex Differences in Regulatory Cells in Experimental Stroke, Cellular Immunology. (2017) 318, 49–54, 10.1016/j.cellimm.2017.06.003, 2-s2.0-85020397859. 28606360 PMC5551457

[32] Cleland N. R. W. , Potter G. J. , and Buck C. , et al.Altered Metabolism and DAM-Signatures in Female Brains and Microglia With Aging, Brain Research. (2024) 1829, 10.1016/j.brainres.2024.148772, 148772. 38244754 PMC12036313

[33] Aryanpour R. , Pasbakhsh P. , and Zibara K. , et al.Progesterone Therapy Induces an M1 to M2 Switch in Microglia Phenotype and Suppresses NLRP3 Inflammasome in a Cuprizone-Induced Demyelination Mouse Model, International Immunopharmacology. (2017) 51, 131–139, 10.1016/j.intimp.2017.08.007, 2-s2.0-85027578373. 28830026

[34] Marriott I. , Bost K. L. , and Huet-Hudson Y. M. , Sexual Dimorphism in Expression of Receptors for Bacterial Lipopolysaccharides in Murine Macrophages: A Possible Mechanism for Gender-Based Differences in Endotoxic Shock Susceptibility, Journal of Reproductive Immunology. (2006) 71, no. 1, 12–27, 10.1016/j.jri.2006.01.004, 2-s2.0-33745640914. 16574244

[35] Kang S. , Ko E. Y. , and Andrews A. E. , et al.Microglia Undergo Sex-Dimorphic Transcriptional and Metabolic Rewiring During Aging, Journal of Neuroinflammation. (2024) 21, no. 1, 2024–2150, 10.1186/s12974-024-03130-7. PMC11155174 38840206

[36] Murray P. J. , Macrophage Polarization, Annual Review of Physiology. (2017) 79, no. 1, 541–566, 10.1146/annurev-physiol-022516-034339, 2-s2.0-85013135313. 27813830

[37] Ginhoux F. , Schultze J. L. , and Murray P. J. , et al.New Insights Into the Multidimensional Concept of Macrophage Ontogeny, Activation and Function, Nature Immunology. (2016) 17, no. 1, 34–40, 10.1038/ni.3324, 2-s2.0-84951335282. 26681460

[38] Park M. D. , Silvin A. , and Ginhoux F. , et al.Macrophages in Health and Disease, Cell. (2022) 185, no. 23, 4259–4279, 10.1016/j.cell.2022.10.007. 36368305 PMC9908006

[39] Martinez F. O. and Gordon S. , The M1 and M2 Paradigm of Macrophage Activation: Time for Reassessment, F1000Prime Reports. (2014) 6, 10.12703/P6-13, 2-s2.0-84897556094, 13. 24669294 PMC3944738

[40] Murray P. J. , Allen J. E. , and Biswas S. K. , et al.Macrophage Activation and Polarization: Nomenclature and Experimental Guidelines, Immunity. (2014) 41, no. 1, 14–20, 10.1016/j.immuni.2014.06.008, 2-s2.0-84904394690. 25035950 PMC4123412

[41] He W. F. and Yan L. F. , [the Regulatory Role and Related Mechanisms of Macrophages in Wound Healing], Zhonghua shao shang yu chuang mian xiu fu za zhi. (2023) 39, no. 2, 106–113, 10.3760/cma.j.cn501225-20230110-00010. 36878519 PMC11630181

[42] He Y. , Gao Y. , and Zhang Q. , et al.IL-4 Switches Microglia/Macrophage M1/M2 Polarization and Alleviates Neurological Damage by Modulating the JAK1/STAT6 Pathway Following ICH, Neuroscience. (2020) 437, 161–171, 10.1016/j.neuroscience.2020.03.008. 32224230

[43] Gong D. , Shi W. , and Yi S. J. , et al.TGFβ Signaling Plays a Critical Role in Promoting Alternative Macrophage Activation, BMC Immunology. (2012) 13, no. 1, 10.1186/1471-2172-13-31, 2-s2.0-84862180623, 31. 22703233 PMC3406960

[44] Liu Y. , Xue M. , and Han Y. , et al.Exosomes From M2c Macrophages Alleviate Intervertebral Disc Degeneration by Promoting Synthesis of the Extracellular Matrix via MiR-124/CILP/TGF-β, Bioengineering & Translational Medicine. (2023) 8, no. 6, 10.1002/btm2.10500, e10500. 38023721 PMC10658595

[45] Konishi H. and Kiyama H. , Microglial TREM2/DAP12 Signaling: A Double-Edged Sword in Neural Diseases, Frontiers in Cellular Neuroscience. (2018) 12, 10.3389/fncel.2018.00206, 2-s2.0-85053332281, 206. 30127720 PMC6087757

[46] Wang X. , Wang Y. , and Yang L. , et al.TREM2(+) Macrophages: A Key Role in Disease Development, Frontiers in Immunology. (2025) 16, 10.3389/fimmu.2025.1550893, 1550893. 40242752 PMC12000036

[47] Carbajosa G. , Malki K. , and Lawless N. , et al.Loss of Trem2 in Microglia Leads to WidespRead Disruption of Cell Coexpression Networks in Mouse Brain, Neurobiology of Aging. (2018) 69, 151–166, 10.1016/j.neurobiolaging.2018.04.019, 2-s2.0-85048526158. 29906661 PMC6075941

[48] Gong N. , Wang W. , and Fu Y. , et al.The Crucial Role of Metabolic Reprogramming in Driving Macrophage Conversion in Kidney Disease, Cellular & Molecular Biology Letters. (2025) 30, no. 1, 10.1186/s11658-025-00746-2, 72. 40524149 PMC12172235

[49] Zhang D. , Tang Z. , and Huang H. , et al.Metabolic Regulation of Gene Expression by Histone Lactylation, Nature. (2019) 574, no. 7779, 575–580, 10.1038/s41586-019-1678-1, 2-s2.0-85074093665. 31645732 PMC6818755

[50] Xu Y. , Meng W. , and Dai Y. , et al.Anaerobic Metabolism Promotes Breast Cancer Survival via Histone-3 Lysine-18 Lactylation Mediating PPARD Axis, Cell Death Discovery. (2025) 11, no. 1, 10.1038/s41420-025-02334-x, 54. 39922804 PMC11807217

[51] Yu X. , Yang J. , and Xu J. , et al.Histone Lactylation: From Tumor Lactate Metabolism to Epigenetic Regulation, International Journal of Biological Sciences. (2024) 20, no. 5, 1833–1854, 10.7150/ijbs.91492. 38481814 PMC10929197

[52] Zeng J. , Bao T. , and Yang K. , et al.The Mechanism of Microglia-Mediated Immune Inflammation in Ischemic Stroke and the Role of Natural Botanical Components in Regulating Microglia: A Review, Frontiers in Immunology. (2023) 13, 10.3389/fimmu.2022.1047550, 1047550. 36818470 PMC9933144

[53] Kojima Y. , Volkmer J. P. , and McKenna K. , et al.CD47-Blocking Antibodies Restore Phagocytosis and Prevent Atherosclerosis, Nature. (2016) 536, no. 7614, 86–90, 10.1038/nature18935, 2-s2.0-84982806125. 27437576 PMC4980260

[54] Green D. R. , Oguin T. H. , and Martinez J. , The Clearance of Dying Cells: Table for Two, Cell Death and Differentiation. (2016) 23, no. 6, 915–926, 10.1038/cdd.2015.172, 2-s2.0-84961223595. 26990661 PMC4987729

[55] Davalos D. , Grutzendler J. , and Yang G. , et al.ATP Mediates Rapid Microglial Response to Local Brain Injury In Vivo, Nature Neuroscience. (2005) 8, no. 6, 752–758, 10.1038/nn1472, 2-s2.0-22244464662. 15895084

[56] Jiang C. T. , Wu W. F. , and Deng Y. H. , et al.Modulators of Microglia Activation and Polarization in Ischemic Stroke (Review), Molecular Medicine Reports. (2020) 21, 2006–2018, 10.3892/mmr.2020.11003. 32323760 PMC7115206

[57] Kim E. , Yang J. , and Beltran C. D. , et al.Role of Spleen-Derived Monocytes/Macrophages in Acute Ischemic Brain Injury, Journal of Cerebral Blood Flow and Metabolism. (2014) 34, no. 8, 1411–1419, 10.1038/jcbfm.2014.101, 2-s2.0-84905508640. 24865998 PMC4126087

[58] Fann D. Y. , Lee S. Y. , and Manzanero S. , et al.Intravenous Immunoglobulin Suppresses NLRP1 and NLRP3 Inflammasome-Mediated Neuronal Death in Ischemic Stroke, Cell Death & Disease. (2013) 4, no. 9, 10.1038/cddis.2013.326, 2-s2.0-84884993094, e790. 24008734 PMC3789184

[59] Gong J. , Gao X. , and Ge S. , et al.The Role of cGAS-STING Signalling in Metabolic Diseases: From Signalling Networks to Targeted Intervention, International Journal of Biological Sciences. (2024) 20, no. 1, 152–174, 10.7150/ijbs.84890. 38164186 PMC10750282

[60] Yan J. , Li A. , and Chen X. , et al.Glycolysis Inhibition Ameliorates Brain Injury After Ischemic Stroke by Promoting the Function of Myeloid-Derived Suppressor Cells, Pharmacological Research. (2022) 179, 10.1016/j.phrs.2022.106208, 106208. 35398239 PMC10364470

[61] Chaitanya G. V. , Eeka P. , and Munker R. , et al.Role of Cytotoxic Protease Granzyme-b in Neuronal Degeneration During Human Stroke, Brain Pathology. (2011) 21, no. 1, 16–30, 10.1111/j.1750-3639.2010.00426.x, 2-s2.0-78649982098. 20825413 PMC8094313

[62] Bastos J. , O’Brien C. , and Vara-Pérez M. , et al.Monocytes Can Efficiently Replace All Brain Macrophages and Fetal Liver Monocytes Can Generate Bona Fide SALL1(+) Microglia, Immunity. (2025) 58, no. 5, 1269–1288.e1212, 10.1016/j.immuni.2025.04.006. 40311613 PMC12094688

[63] Berghoff S. A. , Spieth L. , and Saher G. , Local Cholesterol Metabolism Orchestrates Remyelination, Trends in Neurosciences. (2022) 45, no. 4, 272–283, 10.1016/j.tins.2022.01.001. 35153084

[64] Berghoff S. A. , Spieth L. , and Sun T. , et al.Microglia Facilitate Repair of Demyelinated Lesions via Post-Squalene Sterol Synthesis, Nature Neuroscience. (2021) 24, no. 1, 47–60, 10.1038/s41593-020-00757-6. 33349711 PMC7116742

[65] Kaffe E. , Fiorotto R. , and Pellegrino F. , et al.β-Catenin and Interleukin-1β-Dependent Chemokine (C-X-C Motif) Ligand 10 Production Drives Progression of Disease in a Mouse Model of Congenital Hepatic Fibrosis, Hepatology. (2018) 67, no. 5, 1903–1919, 10.1002/hep.29652, 2-s2.0-85044379397. 29140564 PMC5906178

[66] Fang S. , Xu M. , and Cao L. , et al.Stereopy: Modeling Comparative and Spatiotemporal Cellular Heterogeneity via Multi-Sample Spatial Transcriptomics, Nature Communications. (2025) 16, no. 1, 10.1038/s41467-025-58079-9, 3741. PMC12012134 40258830

[67] Zhang R. , Wu Y. , and Xie F. , et al.RGMa Mediates Reactive Astrogliosis and Glial Scar Formation Through TGFβ1/Smad2/3 Signaling After Stroke, Cell Death and Differentiation. (2018) 25, no. 8, 1503–1516, 10.1038/s41418-018-0058-y, 2-s2.0-85041570158. 29396549 PMC6113216

[68] Baixauli-Martín J. , Burguete M. C. , and López-Morales M. A. , et al.Spatio-Temporal Characterization of Cellular Senescence Hallmarks in Experimental Ischemic Stroke, International Journal of Molecular Sciences. (2025) 26, no. 5, 10.3390/ijms26052364, 2364. 40076983 PMC11900039

[69] Yu S. , Fu J. , and Wang J. , et al.The Influence of Mitochondrial-DNA-Driven Inflammation Pathways on Macrophage Polarization: A New Perspective for Targeted Immunometabolic Therapy in Cerebral Ischemia-Reperfusion Injury, International Journal of Molecular Sciences. (2022) 23, no. 1, 10.3390/ijms23010135, 135. PMC8745401 35008558

[70] Choi Y. H. , Laaker C. , and Hsu M. , et al.Molecular Mechanisms of Neuroimmune Crosstalk in the Pathogenesis of Stroke, International Journal of Molecular Sciences. (2021) 22, no. 17, 10.3390/ijms22179486, 9486. 34502395 PMC8431165

[71] Mokbel A. Y. , Burns M. P. , and Main B. S. , The Contribution of the Meningeal Immune Interface to Neuroinflammation in Traumatic Brain Injury, Journal of Neuroinflammation. (2024) 21, no. 1, 2024–2135, 10.1186/s12974-024-03122-7. PMC11131220 38802931

[72] Yamaga S. , Aziz M. , and Murao A. , et al.DAMPs and Radiation Injury, Frontiers in Immunology. (2024) 15, 10.3389/fimmu.2024.1353990, 1353990. 38333215 PMC10850293

[73] Zheng H. , Liu Q. , and Zhou S. , et al.Role and Therapeutic Targets of P2X7 Receptors in Neurodegenerative Diseases, Frontiers in Immunology. (2024) 15, 10.3389/fimmu.2024.1345625, 1345625. 38370420 PMC10869479

[74] Zhang W. , Zhao J. , and Wang R. , et al.Macrophages Reprogram After Ischemic Stroke and Promote Efferocytosis and Inflammation Resolution in the Mouse Brain, CNS Neuroscience & Therapeutics. (2019) 25, no. 12, 1329–1342, 10.1111/cns.13256. 31697040 PMC6887920

[75] Colonna M. , The Biology of TREM Receptors, Nature Reviews: Immunology. (2023) 23, no. 9, 580–594, 10.1038/s41577-023-00837-1. PMC9904274 36750615

[76] Zhang S. , Li R. , and Song M. , et al.Exploration of M2 Macrophage Membrane as a Biotherapeutic Agent and Strong Synergistic Therapeutic Effects in Ischemic Stroke, Journal of Controlled Release. (2025) 378, 476–489, 10.1016/j.jconrel.2024.11.033. 39561947

[77] Fabre T. , Barron A. M. S. , and Christensen S. M. , et al.Identification of a Broadly Fibrogenic Macrophage Subset Induced by Type 3 Inflammation, Science Immunology. (2023) 8, no. 82, 10.1126/sciimmunol.add8945, eadd8945. 37027478

[78] Ayazi M. , Zivkovic S. , and Hammel G. , et al.Fibrotic Scar in CNS Injuries: From the Cellular Origins of Fibroblasts to the Molecular Processes of Fibrotic Scar Formation, Cells. (2022) 11, no. 15, 10.3390/cells11152371, 2371. 35954214 PMC9367779

[79] Anttila J. E. , Mattila O. S. , and Liew H. K. , et al.MANF Protein Expression Is Upregulated in Immune Cells in the Ischemic Human Brain and Systemic Recombinant MANF Delivery in Rat Ischemic Stroke Model Demonstrates Anti-Inflammatory Effects, Acta Neuropathologica Communications. (2024) 12, no. 1, 10.1186/s40478-023-01701-y, 10. 38229173 PMC10792833

[80] Anttila J. E. , Albert K. , and Wires E. S. , et al.Post-Stroke Intranasal (+)-Naloxone Delivery Reduces Microglial Activation and Improves Behavioral Recovery From Ischemic Injury, Eneuro. (2018) 5, no. 2, 10.1523/ENEURO.0395-17.2018, 2-s2.0-85046426197. PMC5952324 29766045

[81] Ziegler-Heitbrock L. , Monocyte Subsets in Man and Other Species, Cellular Immunology. (2014) 289, no. 2, 135–139, 10.1016/j.cellimm.2014.03.019, 2-s2.0-84899851440. 24791698

[82] Sommer C. J. , Ischemic Stroke: Experimental Models and Reality, Acta Neuropathologica. (2017) 133, no. 2, 245–261, 10.1007/s00401-017-1667-0, 2-s2.0-85008479961. 28064357 PMC5250659

[83] Buscemi L. , Price M. , and Bezzi P. , et al.Spatio-Temporal Overview of Neuroinflammation in an Experimental Mouse Stroke Model, Scientific Reports. (2019) 9, no. 1, 2019–2507, 10.1038/s41598-018-36598-4, 2-s2.0-85060511082. 30679481 PMC6345915

[84] Kato J. , Murata Y. , and Takashima I. , et al.Time- and Area-Dependent Macrophage/Microglial Responses After Focal Infarction of the Macaque Internal Capsule, Neuroscience Research. (2021) 170, 350–359, 10.1016/j.neures.2020.12.001. 33333087

