# Supplementary material for: Spatiotemporal Double‐Edged Sword of Macrophages: Temporal Regulation of Neuroinflammation and Neurorepair in Ischemic Stroke
Source: J Immunol Res. 2026 May 3;2026:1172895. doi: 10.1155/jimr/1172895 (PMC13136591; doi:10.1155/jimr/1172895)
Supplement: Supplementary file 1 — Supporting Information Method This review retrieved literature published from January 2000 to December 2025 by searching the PubMed, Web of Science and Embase databases. The search keywords included: “macrophage”, “microglia”, “stroke”, “cerebral ischemia”, “inflammation”, “neuroinflammation”, “M1/M2 polarization”. Inclusion criteria: (1) Peer‐reviewed English journal articles; (2) In vivo or in vitro studies related to the functions of post‐stroke macrophages and microglia; (3) Studies providing mechanistic insights into macrophage polarization, spatiotemporal dynamics, or functional roles. Exclusion criteria: (1) Abstracts of conferences, editorials and reviews; (2) Non‐English literature; (3) Literature whose full text could not be obtained; (4) Studies focusing exclusively on hemorrhagic stroke without ischemic components. Finally, all included literature was qualitatively and comprehensively analyzed based on thematic relevance. [file JIMR-2026-1172895-s001.docx]

Method

This review retrieved literature published from January 2000 to December 2025 by searching the PubMed, Web of Science and Embase databases. The search keywords included: "macrophage", "microglia", "stroke", "cerebral ischemia", "inflammation", "neuroinflammation", "M1/M2 polarization". Inclusion criteria: (1) Peer-reviewed English journal articles; (2) In vivo or in vitro studies related to the functions of post-stroke macrophages and microglia; (3) Studies providing mechanistic insights into macrophage polarization, spatiotemporal dynamics, or functional roles. Exclusion criteria: (1) Abstracts of conferences, editorials and reviews; (2) Non-English literature; (3) Literature whose full text could not be obtained; (4) Studies focusing exclusively on hemorrhagic stroke without ischemic components. Finally, all included literature was qualitatively and comprehensively analyzed based on thematic relevance.
